# Caloric Restriction Alters the Metabolic Response to a Mixed-Meal: Results from a Randomized, Controlled Trial

**DOI:** 10.1371/journal.pone.0028190

**Published:** 2012-04-16

**Authors:** Kim M. Huffman, Leanne M. Redman, Lawrence R. Landerman, Carl F. Pieper, Robert D. Stevens, Michael J. Muehlbauer, Brett R. Wenner, James R. Bain, Virginia B. Kraus, Christopher B. Newgard, Eric Ravussin, William E. Kraus

**Affiliations:** 1 Physical Medicine and Rehabilitation, Veterans Affairs Medical Center, Durham, North Carolina, United States of America; 2 Division of Rheumatology, Department of Medicine, Duke University Medical Center, Durham, North Carolina, United States of America; 3 Center for Aging and Human Development, Duke University Medical Center, Durham, North Carolina, United States of America; 4 Pennington Biomedical Research Center, Baton Rouge, Louisiana, United States of America; 5 Sarah W. Stedman Nutrition and Metabolism Center and Departments of Pharmacology and Cancer Biology and Medicine, Duke University Medical Center, Durham, North Carolina, United States of America; 6 Division of Cardiovascular Medicine, Department of Medicine, Duke University Medical Center, Durham, North Carolina, United States of America; Paris Institute of Technology for Life, Food and Environmental Sciences, France

## Abstract

**Objectives:**

To determine if caloric restriction (CR) would cause changes in plasma metabolic intermediates in response to a mixed meal, suggestive of changes in the capacity to adapt fuel oxidation to fuel availability or metabolic flexibility, and to determine how any such changes relate to insulin sensitivity (S_I_).

**Methods:**

Forty-six volunteers were randomized to a weight maintenance diet (Control), 25% CR, or 12.5% CR plus 12.5% energy deficit from structured aerobic exercise (CR+EX), or a liquid calorie diet (890 kcal/d until 15% reduction in body weight)for six months. Fasting and postprandial plasma samples were obtained at baseline, three, and six months. A targeted mass spectrometry-based platform was used to measure concentrations of individual free fatty acids (FFA), amino acids (AA), and acylcarnitines (AC). S_I_ was measured with an intravenous glucose tolerance test.

**Results:**

Over three and six months, there were significantly larger differences in fasting-to-postprandial (FPP) concentrations of medium and long chain AC (byproducts of FA oxidation) in the CR relative to Control and a tendency for the same in CR+EX (CR-3 month P = 0.02; CR-6 month P = 0.002; CR+EX-3 month P = 0.09; CR+EX-6 month P = 0.08). After three months of CR, there was a trend towards a larger difference in FPP FFA concentrations (P = 0.07; CR-3 month P = 0.08). Time-varying differences in FPP concentrations of AC and AA were independently related to time-varying S_I_ (P<0.05 for both).

**Conclusions:**

Based on changes in intermediates of FA oxidation following a food challenge, CR imparted improvements in metabolic flexibility that correlated with improvements in S_I_.

**Trial Registration:**

ClinicalTrials.gov NCT00099151

## Introduction

Caloric restriction provides metabolic benefits in a variety of nonhuman animal species (reviewed in [1]). There is great interest in determining whether these metabolic benefits translate to humans and whether caloric restriction induces favorable changes in metabolic indicators of enhanced longevity. The NIH-sponsored Comprehensive Assessment of the Long Term Effects of Reducing Intake of Energy (CALERIE) was a Phase I pilot study that examined the potential health benefits of caloric restriction in sedentary, non-obese, healthy individuals. While the primary aim of the original study was to determine the impact of caloric restriction on biomarkers of longevity and metabolic adaptation, the secondary aims of CALERIE were to evaluate the impact of caloric restriction on risk factors for type 2 diabetes mellitus and cardiovascular disease. In this analysis of data from the Pennington-site CALERIE, we sought to understand if caloric restriction could improve metabolic flexibility.

Metabolic flexibility is an emerging indicator of (metabolic) health [2], [3]. In its most general sense, metabolic flexibility refers to the efficient variation of energy substrate utilization depending on substrate availability and energy demand, or the capacity to adapt fuel oxidation to fuel availability [3], and this is how we interpret the term in this report. To preserve available glucose for use by the brain in the fasting state, most body organs oxidize free fatty acids and amino acids, released through lipolysis and proteolysis, respectively. After a balanced mixed meal, glucose is oxidized in the brain and other organs while lipolysis in adipose tissue and glycogenolysis in the liver are inhibited by increases in insulin levels induced by the ingested calories. Excess glucose is stored as glycogen in the liver and skeletal muscle, and ingested lipids are stored as fat, preferentially in adipose tissue. Efficient substrate switching after a mixed meal manifests as a decrease in fatty acid oxidation and a fall in circulating free fatty acids and intermediates of fatty acid oxidation [2], [3]. Impairment in this normal mode of substrate switching is associated with obesity, skeletal muscle insulin resistance, metabolic syndrome, and type 2 diabetes mellitus [3], [4], [5].

Prior studies have examined the effects of type 2 diabetes, obesity, insulin resistance, and a family history of type 2 diabetes on metabolic flexibility, defined as the ability to shift substrate oxidation and measured by whole body and skeletal muscle respiratory quotient (RQ) [3]. Such investigations have evaluated RQ changes in response to a hyperinsulinemic clamp, high carbohydrate or high fat meals, and high carbohydrate or high fat diets [3].

Thus far in human studies, metabolic flexibility has been measured as alterations in RQ, which serves as a surrogate for substrate oxidation and ranges from 1 for total carbohydrate oxidation to 0.7 for total fat oxidation^3^. Using changes in RQ as the primary variable, weight loss (with and without exercise training) has been associated with a trend towards increased metabolic flexibility, measured in the fasting state alone or in response to a hyperinsulinemic clamp [6], [7], [8]. However, in the setting of an intervention where a mixed meal is delivered, we don not expect RQ to change enough to detect in a heterogenous population. In the current study, we propose that measurement of a broad panel of metabolites that serve as substrates and products of key metabolic pathways can potentially provide a more comprehensive snapshot of changes in metabolic fuel selection in the fasted/fed transition. This concept has been demonstrated in animal studies [9], but not in humans. Derived primarily from intermediate steps in fatty acid oxidation, a rise in plasma levels of even chain acylcarnitines could reflect increased substrate availability for beta oxidation (complete or incomplete) and/or imposition of a shift in flux limitations. Elevated circulating fatty acids might reflect a block in beta oxidation and/or increased lipolysis. Similarly, amino acid concentration increases might reflect a decrease in amino acid use for protein synthesis or as an energy substrate, or an increase in protein turnover.

The main objective of the current study was to determine the effect of caloric restriction on the shift in substrate utilization in response to a mixed meal. A secondary aim was to evaluate the relationship of changes in metabolic flexibility, as measured by metabolic intermediate concentration changes from the fasted to fed states, to changes in insulin sensitivity over the course of a six-month intervention period. We hypothesized that caloric restriction, or the combination of caloric restriction and exercise, would cause a larger difference in the fasting to postprandial concentrations of fatty acids and acylcarnitines over the intervention period when compared to non-caloric restricted controls.

## Methods

### Design and Participants

**Figure 1:**
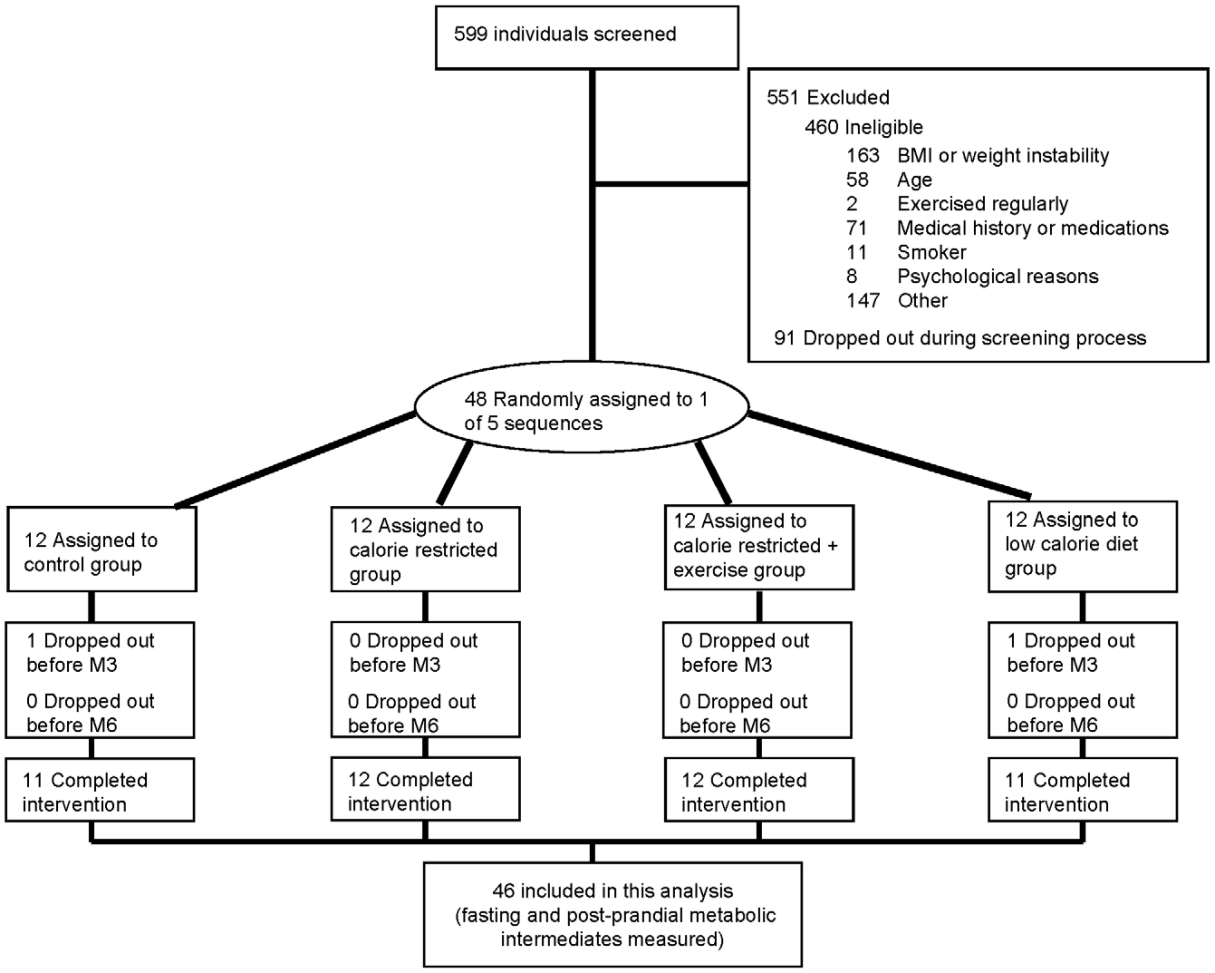
Flow of Participants through the CALERIE trial at Pennington Biomedical Research Center. This Figure has been published previously [10], [25].

Forty-six healthy, non-smoking, overweight (25≤BMI<30), 25–50 year old men and 25–45 year old women were recruited to participate in and completed a six-month intervention (clinical trials number NCT00427193). The protocol for this trial and supporting CONSORT checklist are available as supporting information; see Checklist S1 and Protocol S1. Participants were excluded if they had a history of cardiovascular disease, elevated blood pressure (>140/90 mmHg), high fasting blood glucose (>126 mg/dL), chronic medications (except oral contraceptives), smoking, regular exercise (more than twice a week), abnormal thyroid function or abnormal ECG. Participant flow in the trial is shown in Figure 1.

### Ethics Statement

The study was approved by the Pennington Biomedical Research Center (PBRC, Baton Rouge, LA) Institutional Review Board and the CALERIE Data Safety Monitoring Board, and written informed consent was obtained for all participants.

### Interventions

Participants were randomized into one of four groups for 24 weeks: healthy weight maintenance diet (Control); caloric restriction (CR): 25% caloric restriction from baseline energy requirements; caloric restriction and exercise (CR+EX): 12.5% caloric restriction and 12.5% increase in energy expenditure through structured aerobic exercise; or, liquid calorie diet (LCD; 890 kcal/day) to rapidly achieve 15% weight loss followed by weight stabilization with a eucaloric diet designed to maintain body mass at this level. The group assignment was stratified to ensure equal distributions of sex and BMI in the four groups, and participants were analyzed as intention-to-treat.

### Diets

As previously described, total energy expenditure measured using doubly labeled water was used for calculation of energy requirements [10]. All diets were based on American Heart Association (AHA) recommendations (30% calories from fat, 15% from protein and 55% from carbohydrate).

### Structured Exercise

Individual exercise prescriptions were based on increasing energy expenditure 12.5% above resting energy expenditure as previously described [10]. Exercise modes included walking, running, and cycling at a frequency of five days per week, with at least three supervised sessions per week. Adherence was assessed with heart rate monitors (Polar S-610).

### Metabolic Profiling

Plasma samples were obtained after an overnight fast and 60 and 90 minutes after a standard lunch mixed meal (postprandial and pooled) on an inpatient research unit at each of three time points: baseline (before initiation of the intervention), three months following initiation of the intervention and six months following initiation of the intervention (study exit). Samples were prepared and stored at −80°C for later analysis.

Targeted mass spectrometry was used to measure concentrations of eight free fatty acids (FFA), 15 amino acids (AA), and 45 acylcarnitines (AC) as described previously [11]. In brief, after methylation with iodomethane and partial purification, FFA concentrations were measured with gas chromatography/mass spectrometry (GC/MS). After methanol precipitation and esterification, AC and AA concentrations were measured with tandem mass spectrometry (MS/MS).

### Insulin sensitivity (S_I_)

As previously reported [12], a frequently sampled intravenous glucose tolerance test (IVGTT) was performed with 300 mg/kg of glucose injected at baseline and 0.03 units/kg of Humulin (insulin) injected at minute 20. Concentrations of insulin (DPC 2000, Diagnostic Product Corporation, Los Angeles, CA) and glucose (Synchron CX7, Beckman-Coulter, Brea, CA) were measured at each sampling time. Using these concentrations, S_I_ was derived using Bergman's Minimal Model [13], [14].

### Data Analysis

Principal components analysis (PCA) was used as a data reduction technique. PCA is commonly used to reduce a large number of observed variables to a smaller number of constructed variables (principal components) that account for the variance in the observed variables [15]. It has been used for data reduction in prior studies of metabolic intermediates [11], [16], [17], [18].

Specifically, we created a fasting-to-postprandial change score for each metabolite at each time point as follows: Change score = postprandial – fasting concentration. Then, PCA was performed using varimax rotation on the baseline change score for each metabolite class such that one PCA change score analysis was performed for each domain of FFAs, ACs, and AAs. The number of components retained for each model was selected to balance parsimony and the total percent of variance explained. Then, component loadings from baseline change PCAs were applied to the fasting to postprandial change scores for the three- and six-month time points normalized to the baseline values, and component scores were generated for each metabolic domain at each time point. This normalization provided a common metric for estimating change in components over time.

To determine whether the intervention affected change in fasting-to-postprandial differences (FPPD) over time, we used mixed models [16] to regress FPPD on time, intervention group and an intervention group-by-time product term. FPPD were based on the principal components described above. For these models, the intervention group-by-time product term indicated whether *changes over time* in FPPD vary with intervention group (versus Control). To account for potential nonlinearity of the time effect, time was modeled as a categorical variable. Thus, the omnibus intervention group x time interaction was calculated on 6 df [(4−1 = 3) groups* (3−1 = 2) time points]. Additionally, we used mixed models to regress time-varying insulin sensitivity (S_I_) on time plus each time-varying FPPD component. The coefficients for each time-varying FPPD component in these models indicated whether (averaged across all time points) the FPPD components were positively or negatively related to S_I_. The pseudo R^2^ statistic was used to determine the between person variance in S_I_ [19].

## Results

Information on the main determinants from CALERIE is reported elsewhere. Briefly, CR and CR+EX improved two biomarkers associated with longevity, reduced core body temperature and fasting insulin concentrations as well as 24-hour energy expenditure, DNA damage, and cardiovascular risk profiles [10], [22]. Also, CR and CR+EX improved weight and fat mass, pancreatic beta cell function, and intrahepatic lipid content [12], [23].

### Fasting to Postprandial Changes in Metabolites

Using PCA to analyze changes in the fasting-to-postprandial difference (FPPD), we identified single components for FPPD for each of the major analyte modules– FFA, AC, and AA– with eigenvalues of 6.37, 8.67, and 7.88 respectively, explaining 80%, 20% and 53% of the variance in the component (Table 1). At the initial assessment, individual FFA and AC metabolites generally decreased from fasting to the postprandial state, particularly those that loaded heavily as part of the FFA and AC components (Table 2). In contrast, individual AA concentrations increased in the transition to the postprandial state (Table 2).

**Table 1:** Principal Components Analyses (PCA) for Fasting to Postprandial Changes in Metabolites.*

Constituents	Loadings	Eigen-value	Cumulative Variance
*Post-Pre Fatty Acid Component 1*			
Palmitic acid	0.96	6.37	0.80
Linoleic acid	0.95		
Oleic acid	0.95		
Myristic acid	0.93		
Palmitoleic acid	0.91		
Stearic acid	0.87		
alpha-Linolenic acid	0.86		
Arachidonic acid	0.66		
*Post-Pre Acylcarnitine Component 1*			
C12:1	0.77	8.67	0.20
C16	0.77		
C14:1	0.75		
C14:2	0.72		
C16:2	0.67		
C16:1	0.67		
C12	0.69		
C18:1	0.66		
C6-DC	0.62		
C10:1	0.61		
C10	0.56		
C8:1	0.54		
C8	0.53		
C10-OH/C8-DC	0.53		
C18:2	0.53		
C8:1-DC	0.51		
*Post-Pre Amino Acid Component 1*			
Leucine/Isoleucine	0.91	7.88	0.53
Phenylalanine	0.91		
Methionine	0.87		
Histidine	0.83		
Valine	0.81		
Tyrosine	0.81		
Aspartate/Asparagine	0.81		
Serine	0.79		
Proline	0.71		
Ornithine	0.66		
Arginine	0.65		
Glycine	0.56		
Alanine	0.56		

*Changes were computed as postprandial metabolite concentration minus preprandial metabolite concentration. For these differences, PCA was performed separately for each metabolite class: fatty acids, acylcarnitines, and amino acids. Key metabolites within each component (*i.e.*, metabolites with component load ≥|0.5|) are presented.

**Table 2:** Median and interquartile range (IQR) of fasting and postprandial concentrations of metabolites at baseline (n = 46).*

Metabolite Changes	Preprandial Median (IQR)	Postprandial Median (IQR)
***Free Fatty Acids***		
**Myristic acid, FA-C14:0 (µM)**	**6.64 (4.21)**	**2.00 (1.82)**
**Palmitoleic acid FA-C16:1(ω-7) (µM)**	**13.19 (11.31)**	**1.41 (1.01)**
**Palmitic acid, FA-C16:0 (µM)**	**110.5 (60.1)**	**22.9 (16.7)**
**α-Linolenic acid, FA-C18:3(ω-3) (µM)**	**5.15 (2.40)**	**1.25 (1.11)**
**Linoleic acid, FA- C18:2(ω-6) (µM)**	**77.4 (53.1)**	**16.5 (10.9)**
**Oleic acid, FA-C18:1(ω-9) (µM)**	**165.7 (64.4)**	**31.9 (26.0)**
**Stearic acid, FA-C18:0 (µM)**	**42.0 (18.0)**	**11.0 (7.4)**
**Arachidonic acid, FA-C20:4(ω-6) (µM)**	**4.18 (1.62)**	**2.41 (1.60)**
***Acylcarnitines***		
Acetyl carnitine, C2 (nM)	6941.3 (3053.4)	4169.4 (1929.1)
Propionyl carnitine, C3 (nM)	386.6 (243.2)	471.9 (273.9)
Butyryl/Isobutyryl carnitine, C4/Ci4 (nM)	136.0 (112.8)	160.3 (64.5)
Tiglyl carnitine, C5:1 (nM)	52.0 (21.6)	48.1 (25.1)
Isovaleryl/3-Methylbutyryl/2-Methylbutyryl carnitine, C5's (nM)	102.0 (54.8)	110.6 (61.0)
β-Hydroxy butyryl carnitine, C4OH (nM)	21.0 (18.0)	16.9 (19.6)
Hexanoyl carnitine, C6 (nM)	0 (0)	21.3 (69.2)
3-Hydroxy-isovaleryl/Malonyl carnitine, C5OH/C3DC (nM)	92.8 (88.0)	104.1 (111.2)
Methylmalonyl/Succinyl carnitine, Ci4DC/C4DC (nM)	21.8 (14.1)	22.6 (13.7)
Octenoyl carnitine, C8:1 (nM)	151.4 (76.3)	122.8 (68.4)
**Octanoyl carnitine, C8 (nM)**	**70.1 (32.5)**	**29.3 (19.1)**
Glutaryl carnitine, C5DC (nM)	28.9 (17.7)	23.0 (13.7)
**Adipoyl carnitine, C6DC (nM)**	**39.4 (29.0)**	**27.6 (18.9)**
Decatrienoyl carnitine, C10:3 (nM)	96.8 (47.7)	71.0 (41.0)
Decadienoyl carnitine, C10:2 (nM)	28.5 (11.7)	21.1 (15.2)
**Decenoyl carnitine, C10:1 (nM)**	**140.5 (67.3)**	**58.8 (29.5)**
**Decanoyl carnitine, C10 (nM)**	**142.1 (72.2)**	**45.4 (74.1)**
**3-Hydroxy-decanoyl/Suberoyl carnitine, C10-OH/C8-DC (nM)**	**14.1 (7.4)**	**6.0 (4.0)**
**Dodecenoyl carnitine, C12:1 (nM)**	**57.2 (17.3)**	**26.2 (12.4)**
**Lauroyl carnitine, C12 (nM)**	**38.6 (23.2)**	**16.2 (10.1)**
**3-Hydroxy-dodecanoyl/Sebacoyl carnitine, C12-OH/C10-DC (nM)**	**4.1 (2.5)**	**1.5 (2.5)**
**Tetradecadienoyl carnitine, C14:2 (nM)**	**23.1 (12.2)**	**6.9 (5.1)**
**Tetradecenoyl carnitine, C14:1 (nM)**	**39.4 (17.7)**	**12.4 (6.0)**
Myristoyl carnitine, C14 (nM)	12.7 (7.4)	8.5 (4.4)
3-Hydroxy-tetradecenoyl carnitine, C14:1-OH (nM)	8.7 (5.3)	6.0 (5.1)
3-Hydroxy-tetradecanoyl/Dodecanedioyl carnitine, C14-OH/C12-DC (nM)	5.6 (3.9)	3.5 (3.2)
**Palmitoyl carnitine, C16 (nM)**	**60.8 (20.4)**	**42.4 (15.6)**
3-Hydroxy-hexadecanoyl/Tetradecanedioyl carnitine, C16-OH/C14-DC (nM)	2.1 (1.9)	2.1 (1.6)
**Linoleyl carnitine, C18:2 (nM)**	**54.1 (18.3)**	**38.1 (21.8)**
**Oleyl carnitine, C18:1 (nM)**	**96.7 (40.3)**	**61.7 (22.1)**
Stearoyl carnitine, C18 (nM)	26.2 (9.0)	23.7 (11.4)
3-Hydroxy-octadecenoyl carnitine, C18:1-OH (nM)	3.9 (1.9)	2.3 (1.8)
3-Hydroxy-octadecanoyl/Hexadecanedioyl carnitine, C18-OH/C16-DC (nM)	3.2 (2.8)	3.0 (1.8)
Arachidoyl carnitine, C20 (nM)	3.5 (2.6)	3.2 (2.5)
Octadecenedioyl carnitine, C18:1-DC (nM)	4.3 (2.3)	4.0 (2.4)
3-Hydroxy-eicosanoyl/Octadecanedioyl carnitine, C20-OH/C18-DC (nM)	4.7 (3.5)	4.1 (3.7)
Docosanoyl carnitine, C22 (nM)	2.2 (1.8)	1.9 (1.6)
3-Hydroxy-*cis*-5-octenoyl/Hexenedioyl carnitine, C8:1-OH/C6:1-DC (nM)	22.1 (12.2)	16.0 (8.0)
Heptanedioyl carnitine, C7-DC (nM)	0 (6.2)	0 (4.9)
**Octenedioyl carnitine, C8:1-DC (nM)**	**13.5 (8.4)**	**12.1 (7.3)**
**Hexadecadienoyl carnitine, C16:2 (nM)**	**5.6 (3.2)**	**2.3 (2.4)**
**Palmitoleoyl carnitine, C16:1 (nM)**	**15.4 (6.1)**	**5.9 (4.6)**
3-Hydroxy-palmitoleoyl/*cis*-5-Tetradecenedioyl carnitine, C16:1-OH/C14:1-DC, (nM)	5.3 (1.9)	3.2 (2.2)
3-Hydroxy-linoleyl carnitine, C18:2-OH (nM)	3.7 (4.3)	2.9 (4.9)
**Arachidonoyl carnitine, C20:4 (nM)**	**6.2 (4.2)**	**5.7 (4.6)**
***Amino Acids***		
**Glycine (µM)**	**359.4 (113.2)**	**382.6 (124.9)**
**Alanine (µM)**	**411.4 (117.8)**	**539.6 (126.2)**
**Serine (µM)**	**140.4 (32.5)**	**151.6 (32.8)**
**Proline (µM)**	**196.2 (64.1)**	**297.9 (55.8)**
**Valine (µM)**	**254.3 (53.8)**	**274.9 (58.2)**
**Leucine/Isoleucine (µM)**	**174.3 (46.5)**	**209.3 (63.4)**
**Methionine (µM)**	**28.7 (5.2)**	**34.0 (5.7)**
**Histidine (µM)**	**109.2 (38.6)**	**118.7 (36.9)**
**Phenylalanine (µM)**	**81.2 (13.9)**	**101.0 (11.0)**
**Tyrosine (µM)**	**68.4 (19.3)**	**81.3 (15.8)**
**Aspartate/Asparagine (µM)**	**34.3 (9.4)**	**36.4 (9.5)**
Glutamate/Glutamine (µM)	102.9 (36.9)	105.6 (40.5)
**Ornithine (µM)**	**69.9 (25.0)**	**90.4 (29.6)**
Citrulline (µM)	30.7 (11.8)	28.4 (10.7)
**Arginine (µM)**	**134.1 (36.4)**	**152.1 (45.5)**

*Those loading most heavily (component load ≥|0.5|) in principal component analyses are identified in bold.

**Figure 2:**
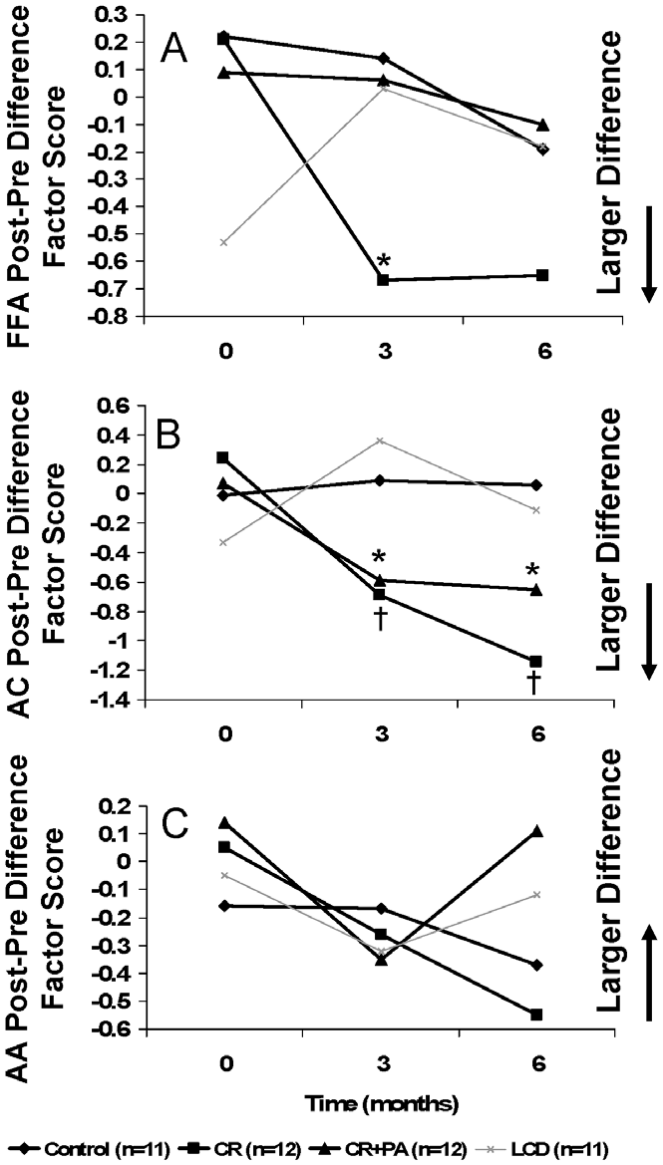
Trajectories of Fasting to Postprandial Metabolic Intermediate Concentration Changes over Time by Intervention Group. Fasting to postprandial difference (FPPD) scores were computed as postprandial minus fasting concentration. Difference scores were used in principal component analyses and single component solutions were retained as described in Results. FPPD component scores in Figures A-C were calculated as: (1) FPPD = S1*(I1)+S2*(I2) +… Sk*(Ik); where: I1-ik are the k individual items used in the principal components analysis; S1–Sk are standardized scoring coefficients from the principal component analysis; and, I1-Ik are entered as (I/SdI) for each item. With equation 1, the FPPD component score is the average of the mean post-preprandial differences across items with each item weighted by its standardized scoring coefficient. Mean difference are expressed in standard deviation units. Significant trends (P<0.10) are identified with an asterisk (*) and significant group by time interactions are indicated with a (†). CR = Caloric restriction. CR+EX = Combined caloric restriction and exercise. LCD = Liquid calorie diet. **A.** Free Fatty Acids (FFA). (CR * 3 months P = 0.07) **B.** Acylcarnitines (AC). (CR * 3 months P = 0.02; CR * 6 month P = 0.002; CR+EX * 3 month P = 0.09; CR+EX * 6 month P = 0.08) **C.** Amino Acids (AA).

In response to CR, the AC FPPD was amplified (more negative FPPD) over time relative to Control (P = 0.02 at 3 mo., P = 0.002 at 6 mo., overall P<0.001) (Figure 2B). A similar trend was observed for the CR+EX group (P = 0.09 at 3 mo., P = 0.08 at 6 mo.; Figure 2B). In response to CR, the FFA FPPD was also amplified (p = 0.08 at 3 mo., p = 0.40 at 6 mo., overall p = 0.07, Figure 2A). In response to both CR and CR+EX, the change in AA FPPD was not significant (overall p = 0.87). In response to LCD, there were no sustained responses in AC, FFA, or AA FPPD (P>0.10 for all). However, the increase in FPPD for AC and FFA in subjects exposed to CR supports the conclusion that these subjects achieved an increase in metabolic flexibility as a consequence of the intervention.

### Relations among S_I_, exercise, and metabolite concentrations

**Figure 3:**
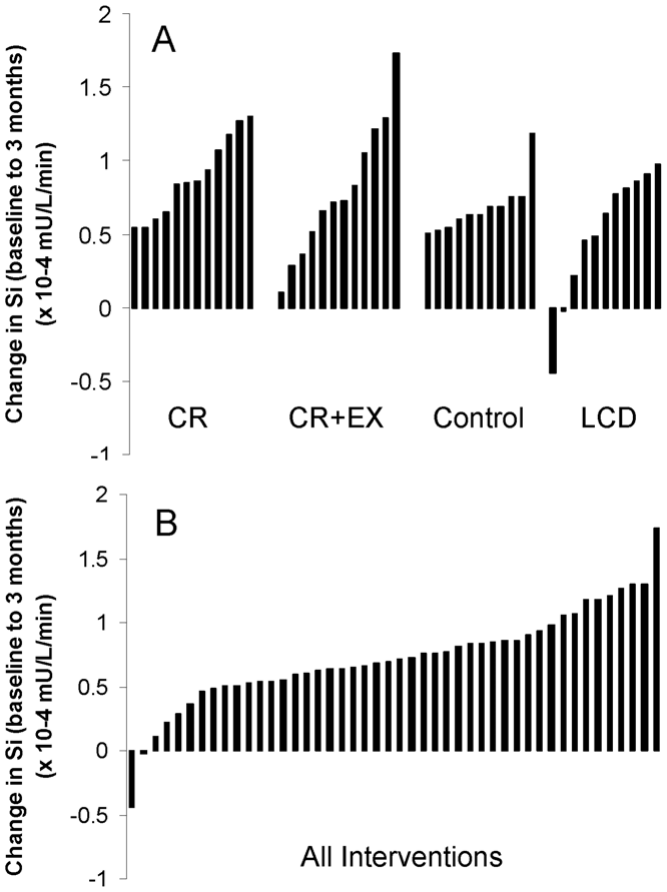
Baseline to Month Three Changes in Insulin Sensitivity: Average Group Improvements Despite Varied Individual Responses. Each bar represents insulin sensitivity improvements for participating individuals. A. By intervention group. CR = Caloric restriction; CR+EX = Caloric restriction with exercise; Control = Healthy weight maintenance diet; LCD = Liquid calorie diet B. Intervention groups combined.

**Figure 4:**
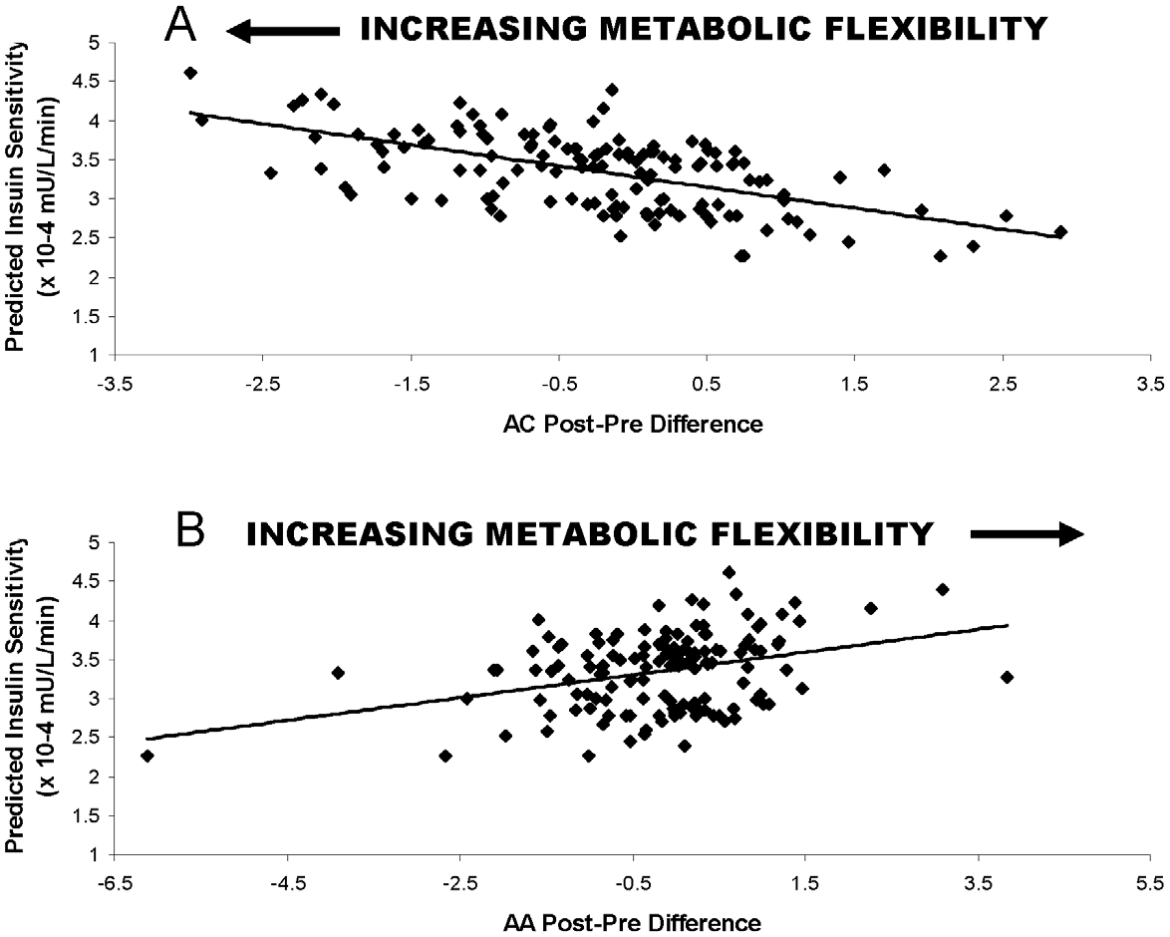
Correlation Between Fasting to Postprandial Component Changes and Predicted S_I_ Change Over Time. As described in Methods, fasting and postprandial concentrations of amino acids and acylcarnitines were measured at baseline, three months, and six months, and fasting to postprandial components were generated. S_I_ was determined from insulin and glucose concentrations measured during a frequently sampled intravenous tolerance test at each of baseline, three, and six months. Linear models were used to relate time varying concentrations of fasting to postprandial amino acid and acylcarnitine component to time varying insulin sensitivity (S_I_). Scatter plots depict the relation between fasting to postprandial component scores and predicted S_I_. A. Relation between Acylcarnitines (AC) Fasting to Postprandial Component Scores and Predicted S_I_ Over Time. Since postprandial AC concentrations are larger than fasting, more negative fasting to postprandial differences represent more metabolic flexibility. B. Relation between Amino Acid (AA) Fasting to Postprandial Component Scores and Predicted S_I_ Over Time.

As previously reported [12], between baseline and six months, there was significant improvement in S_I_ in the CR+EX group and a trend towards improvement in S_I_ in the CR group compared to Control (P = 0.01, P = 0.08, respectively). For each of these treatment groups, we observed a broad range of changes in insulin sensitivity; specifically, despite an average improvement in insulin sensitivity for each group, the individual participant responses were varied (Figure 3A). To better understand how insulin sensitivity improvements related to changes in FPPD, irrespective of how the change in S_I_ was achieved, all subjects were included in these regression analyses ignoring group assignment (Figure 3B). There were no significant relations between FPPD in FFA and time-varying S_I_ at 3 months (P = 0.54) or at six months (P = 0.91). In contrast, in a model that predicted S_I_, given both time-varying AC and AA components, FPPD in AC concentrations were inversely related to time-varying S_I_ (P = 0.04; Figure 4A), and FPPD in AA concentrations were positively related to time-varying S_I_ (P = 0.04; Figure 4B). The AA and AC FPPD scores explained 18% of the between person variance in S_I_. These results indicate that averaged across all time points, higher S_I_ was associated with better metabolic flexibility as represented by larger changes in AC and AA FPPD scores.

**Figure 5:**
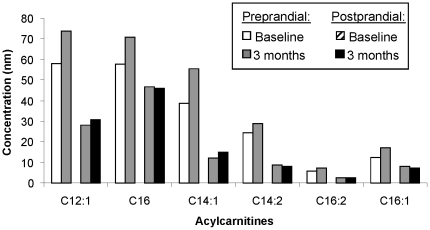
Preprandial and postprandial concentrations of acylcarnitines in response to caloric restriction (CR). Baseline and three month acylcarnitine concentrations are shown for both fasting (preprandial) and postprandial assessments. The six acylcarnitines that had the largest loadings on the acylcarnitine factor (see Table 1) are shown.

**Figure 6:**
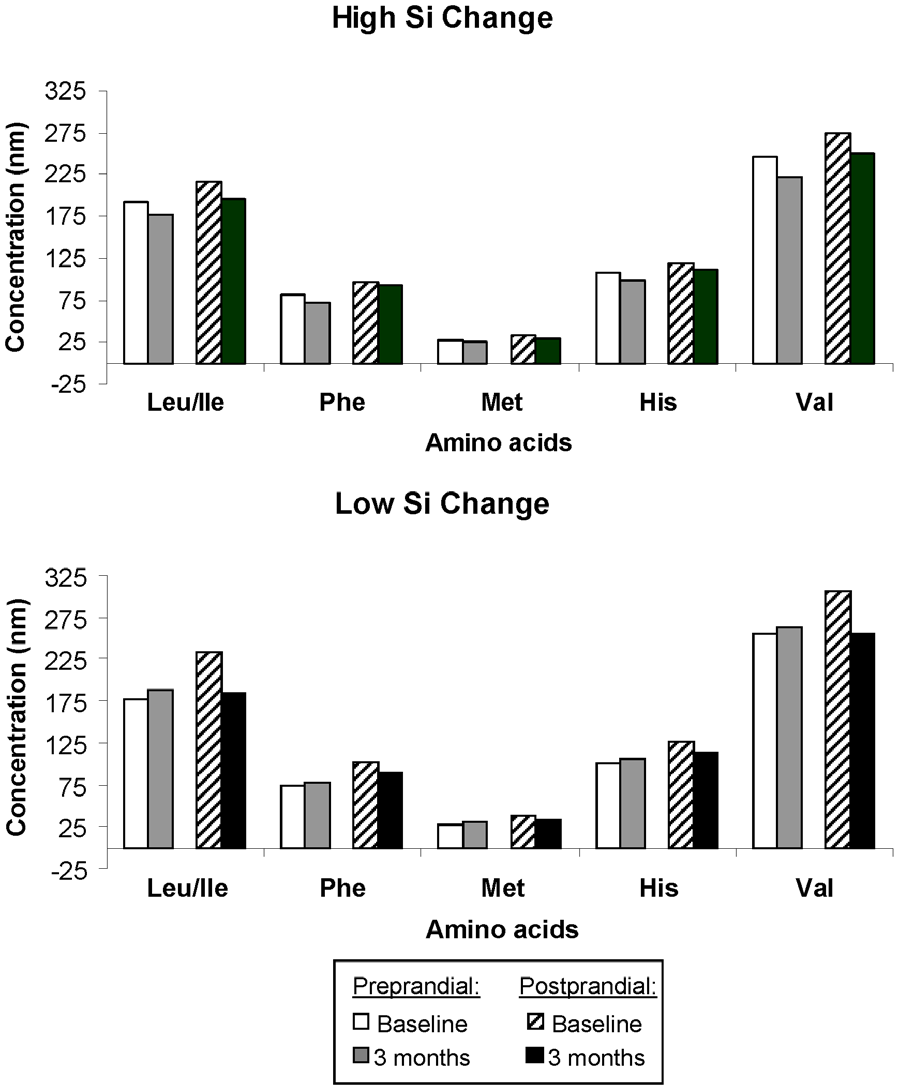
Preprandial and postprandial concentrations of amino acids for those with the highest and lowest insulin sensitivity changes. Baseline and three month amino acids concentrations are shown for both fasting (preprandial) and postprandial assessments. The five amino acids that had the largest loadings on the amino acid factor (see Table 1) are shown. Leu/Ile = leucine/isoleucine, Phe = phenylalanine, Met = Methionine, His = Histidine, Val = Valine.

In order to determine whether larger FPPD corresponded to changes in fasting concentrations, postprandial concentrations or both, we evaluated raw data for individual metabolites loading most heavily on each component. By evaluating raw data, the intervention-induced amplifications in FFPD were attributable to changes in the fasting concentrations, as opposed to the postprandial concentration of the metabolites; that is, increased fasting AC and decreased fasting AA over time. As seen in Figure 5, in response to caloric restriction (CR), fasting AC concentrations increased while postprandial changes were variable. For AAs, despite a reduction in the AA difference in response to CR, larger pre-postprandial differences in AAs were related to higher S_I_s. These observations emphasize that there were not one to one correlations between CR and improvements in S_I_. However, it remained unclear for those with the largest time varying S_I_ whether the pre to postprandial AA differences increased over time because the fasting AA concentrations decreased or the post-prandial concentrations increased. To address this, we evaluated the mean pre and postprandial AAs for participants with the six largest and six smallest predicted S_I_ changes. As seen in Figure 6, for those with high S_I_ changes fasting AA concentrations decreased while for those with low S_I_ changes, fasting AA concentrations increased or remained unchanged. Postprandial AA concentrations decreased over time both in those with the most and least S_I_ improvements. Therefore, for both ACs and AAs, the larger FPPDs arose primarily from changes in fasting concentrations with increases in ACs and decreases in AAs.

## Discussion

In this randomized controlled trial of three healthy lifestyle interventions (CR, CR+EX, and a healthy weight maintenance diet) and a very low calorie diet, we observed that the CR intervention significantly increased the fasting-to-postprandial difference (FPPD) in circulating acylcarnitines (AC) and free fatty acids (FFA). Moreover, we observed that increased FPPD for both AC and AA were related to greater insulin sensitivity. Thus, this study expands prior investigations of metabolic flexibility from 1) acute responses to an infusion or meal to responses to a prolonged intervention, 2) responses to glucose/insulin infusions to more clinically-relevant mixed meals, 3) assessments made by increases in RQ to changes in a broad panel of metabolic intermediates that includes both substrates and products of key energy producing pathways.

The term metabolic flexibility is used to describe the efficient transition between substrate utilization in response to changes in substrate supply or energy demand. One example is the shift from the use of fatty acids as the main energy source during fasting conditions towards glucose utilization in a fed state [3]. Three months of CR led to an increased gradient in pre- to postprandial AC concentrations, primarily due to an increase in fasting AC concentrations. Since AC are intermediates in fatty acid oxidation, increased fasting AC concentrations reflect either a block in complete fatty acid oxidation or increases in lipolysis and fatty acid oxidation. These findings were supported by the concurrent increase in the gradient of pre-postprandial FFA, primarily mediated by increased fasting FFA concentrations. Since these changes occurred in the setting of caloric restriction where energy demand exceeds supply, in the fasting state when fatty acid oxidation predominates, and these changes associate with improved health outcomes, including insulin sensitivity, we believe an increase in fatty acid oxidation is more likely. Thus, these data imply that fatty acids are more effectively mobilized and oxidized in the fasted state in subjects that have undergone CR. The mechanisms underlying these effects remain to be investigated.

To our knowledge, this is the first report of a relation between an increased FPPD for AA and greater insulin sensitivity. While in prior investigations, amplified FPPD gradients in fatty acid and glucose oxidation have been recognized as signs of improved metabolic flexibility [4], [8], there has been little focus on AA metabolism in this context. Our findings here are consistent with our prior studies showing that elevated fasting concentrations of branched chain amino acids (BCAA) and related metabolites are associated with insulin resistance [11], [16]. Interestingly, in the current investigation, BCAA garnered the highest loadings on the AA principal component. Here, we observed that the ability to modulate AA concentrations was associated with improved insulin sensitivity, and this modulation was due primarily to a reduction in fasting AA concentrations. Further, since dietary composition was controlled and maintained constant throughout the investigation, changes in AA concentrations are not likely related to dietary changes. These observations emphasize that the concept of metabolic flexibility should not be limited to discussions of fat and glucose metabolism but should also include AA metabolism.

Here, we showed that CR (at a level of −25% from basal energy requirements) improved the ability to shift energy substrates with feeding, and that caloric restriction plus exercise (CR+EX), with an identical relative energy deficit, imparted roughly half of the ability to shift substrate seen with CR alone. In contrast, in a study by Kelley and Goodpaster, a combination of weight loss and exercise training increased the rate of fatty acid relative to glucose oxidization in the fasting state (lower fasting respiratory quotient [RQ]) as compared to before the intervention [8]; however, in the absence of exercise training, there was little change in fasting RQ with CR alone [4]. We note that our study differs from that of Kelley and Goodpaster in terms of the “feeding” challenge (clamp versus a mixed meal), assessment of substrate oxidation with metabolic intermediates rather than RQ, targeting the transition from the fasted to the fed state in contrast to their emphasis on fasting fat oxidation alone, and differences in exercise regimens or timing of assessments after training cessation [20]. Moreover, Kelley and Goodpaster observed that increased fasting fatty acid oxidation relative to glucose oxidation was highly associated with improvements in S_I_ [8]. While we observed a relationship between the two, we noted that Si improvements were greatest in the CR+EX group, but substrate utilization improvements were greatest in the CR group. We also note that the subjects in the CALERIE study were healthy with normal insulin sensitivity prior to the CR or CR + EX interventions, whereas those studied by Goodpaster and Kelly were insulin resistant [4], [8], [21]. These findings suggest that while improvements in insulin sensitivity are related to metabolic flexibility changes, there are additional components of the variability of each that are yet to be identified.

Our observations add metabolic flexibility, as measured by changes in metabolic intermediates in response to a mixed meal, to the list of improvements that occur in the setting of CR elucidated through the CALERIE study. In addition to this effect, CR or CR+EX reduced core body temperature, 24-hour energy expenditure, fasting insulin concentrations, DNA damage, and cardiovascular risk profiles [10], [22]. Matched for energy deficit, CR and CR+EX produced similar improvements in reductions in weight and fat mass, pancreatic beta cell function, and intrahepatic lipid content [12], [23]. In contrast, while both CR and CR+EX increased adiponectin concentrations, and skeletal muscle sirtuin 1 (SIRT1) expression, these increases were more pronounced in the CR alone group, suggesting that these effects were primarily mediated by the CR component of the combined intervention [24]. Similarly, 25% CR produced a greater metabolic adaptation (as defined by a reduction in energy expenditure) than would be predicted for loss in body mass alone, and that was not observed with 12.5% CR+EX [25].

The careful control of the caloric restriction and exercise interventions is a great strength of this analysis. To minimize type I error rates, we used PCA to reduce the dimensionality of the data. We performed these analyses cognizant that using PCA in small samples can result in ‘overfitting,’ where findings are sample-specific rather than representative of the population of interest [26]. However, the PCA-derived components and loadings here were both substantively credible and consistent with findings from previous studies using PCA on the same or similar metabolite measures [11], [16], [17], [18]. With a sample size of 46, we had limited power to observe small to medium sized effects, and given this, we present statistical trends (0.05<P<0.10) in several instances. Additionally, while the metabolite profiles can identify perturbations in systemic trafficking of specific carbon fuels, they do not provide definitive information about metabolic flux. Nonetheless, the results of such analyses provide a valuable guide for further investigations seeking to identify underlying mechanisms.

In summary, CR improved metabolic flexibility evidenced by higher fasting AC and FFA concentrations and widened FPPD gradients for these metabolites. Furthermore, the change in the FPPD gradient of AC and AA concentration was related to improvements in insulin sensitivity.

## Supporting Information

Checklist S1 CONSORT Checklist.
(DOC)

Protocol S1 Trial Protocol.
(DOC)
